# Trained immunity inducers in cancer immunotherapy

**DOI:** 10.3389/fimmu.2024.1427443

**Published:** 2024-07-16

**Authors:** Yongjun Sui, Jay A. Berzofsky

**Affiliations:** Vaccine Branch, Center for Cancer Research, National Cancer Institute, National Institutes of Health (NIH), Bethesda, MD, United States

**Keywords:** trained immunity, cancer immunotherapy, tumor microenvironment, T cell responses, interferon γ

## Abstract

While most of the cancer immunotherapy strategies engage adaptive immunity, especially tumor-associated T cells, the small fraction of responding patients and types of cancers amenable, and the possibility of severe adverse effects limit its usage. More effective and general interventions are urgently needed. Recently, a de facto innate immune memory, termed ‘trained immunity’, has become a new research focal point, and promises to be a powerful tool for achieving long-term therapeutic benefits against cancers. Trained immunity-inducing agents such as BCG and fungal glucan have been shown to be able to avert the suppressive tumor microenvironment (TME), enhance T cell responses, and eventually lead to tumor regression. Here, we review the current understating of trained immunity induction and highlight the critical roles of emergency granulopoiesis, interferon γ and tissue-specific induction. Preclinical and clinical studies that have exploited trained immunity inducers for cancer immunotherapy are summarized, and repurposed trained immunity inducers from other fields are proposed. We also outline the challenges and opportunities for trained immunity in future cancer immunotherapies. We envisage that more effective cancer vaccines will combine the induction of trained immunity with T cell therapies.

## 1 Introduction

Trained immunity, also called trained innate immunity, is an emerging concept that innate cells harbor features of immunological memory and display enhanced long-term functionality (1–3). In the past decade, both myeloid (monocytes, macrophages and dendritic cells) and lymphoid (natural killer cells and innate lymphoid cells) cells of the innate immune system, as well as non-immune cells (epithelial, stromal cells, fibroblasts and muscle cells) have been shown to display long-term memory upon stimulation (4, 5). Trained immunity bridges innate and adaptive immunity. Its two main features are its antigen non-specific manner and long-term memory. The definition and key features of trained immunity in health and disease has been discussed in reference 3. As suggested by its name, trained innate immunity is mediated by innate cells. Given the fact that innate cells lack antigen-specific immune responses, one hallmark of trained immunity is that it acts in an antigen-independent manner, i.e., it can be evoked by one stimulus and protect the host against a wide range of insults in addition to the original one that induced it. The concept of applying trained immunity in cancer immunotherapy is to apply trained immunity inducers in cancer patients to elicit immune responses that kill tumor cells. The involvement of innate immune and non-immunes cells by this strategy enriches the responding repertoire, and their interplay with adaptive immunity also re-shapes the anti-cancer immunity. Considering that the tumor cells have developed various of escape mechanisms to avoid antigen-specific T cell immune surveillance, the antigen-agnostic attribute of trained immunity may have advantages. We hypothesize that the antigen non-specific manner of trained immunity bypasses the need for cancer antigens and applies to a wider range of cancers. The tumor microenvironment (TME) is composed of suppressive myeloid cells, Treg cells and other innate cells that prevent the function of tumor-infiltrating T cells, while other innate factors and tumor vascular endothelial cells can prevent penetration of T cells into the tumors. One interesting hypothesis is that induction of trained immunity might facilitate the maintenance of durable immune stimulation status in the TME, which alone or combined with T cell therapies might enhance T-cell responses to mediate protection against tumor growth, and avert the re-establishment of favorable metastatic environments to facilitate the long-term prevention of metastasis (6).

## 2 Mechanisms of trained immunity induction and maintenance

Trained immunity is a term to describe innate cells that have memory, which can be initiated *in vivo* by a wide range of stimuli such as BCG or fungal glucan. Deciphering the induction and maintenance of innate memory is critical for the development of new approaches for cancer immunotherapy. The establishment of trained immunity is usually through transcriptomic (RNA expression), metabolic and epigenetic programming (7). Note that the earlier concept that innate cells did not have memory was based on the idea that memory had to be antigen-specific and was mediated in adaptive immune cells by clonal expansion of antigen-specific clones. Without such clonal selection, how could innate cells have memory? However, it is now recognized that both adaptive and innate cells can have memory within a single cell, not requiring clonal expansion. Memory cells of both innate and adaptive immunity change from their naïve state through epigenetic and metabolic changes that apply equally to both. Upon stimulation, at the epigenetic level, DNA methylation, histone modifications, and non-coding RNA regulate chromatin accessibility, and control the gene transcription of both innate and adaptive immune cells (8), while at the metabolic level, increased glycolysis and decreased oxidative phosphorylation lead to faster ATP production and enhanced biosynthesis on both types of cells (9). Thus, at a single cell level, a cell can have a similar intracellular memory phenotype whether it is adaptive or innate. This contrasts with clonal expansion of antigen-specific cells which adds to memory at a cell population level, which can apply only to adaptive antigen-specific immune cells.

Metabolically, aerobic glycolysis, which is mediated through the hypoxia-inducible factor-1α (HIF-1α)-induced mammalian/mechanistic target of rapamycin (mTOR) pathway, and enhanced cholesterol synthesis is a well-established mechanism for inducing trained immunity (10, 11). For transcriptomic (RNA expression) and epigenetic programming, two stages, training and silent stages, are commonly involved. During the training stage, both gene transcriptional and epigenetic alterations are induced by the training agents. At the silent stage, when the training agents are removed or cleared by the host, gene transcription returns to basal levels, while the epigenetic alterations persist for a longer time. The persistent epigenetic alterations make the chromatin in an “open” status, and thus more readily and easily accessible by transcription factors. Upon second homologous or heterologous challenges, the gene transcription is enhanced at a much higher level and in a faster manner (3). As this topic has been extensively reviewed (2, 3), here we focus on the recent understanding of the maintenance of long-term memory in innate cells.

### 2.1 Emergency granulopoiesis and bone marrow imprinting

The immunological memory for trained immunity has been found to last at least months and up to one year (12, 13). However, the lifespan of innate cells in the bloodstream is short. For examples, neutrophils and myeloid cells live 3–7 days. Thus, more studies are focusing on how and where the innate cells retain their memory status. One mechanism is through the imprinting of long-lived hematopoietic stem and progenitor cell (HSPC). Mitroulis et al. found that the HSPC engagement is essential for the long-term maintenance of the trained immunity (14). Since the epigenetic programming occurs in the bone marrow progenitor cell, their progeny cells carry on the same feature thereafter. This is in line with the recent observation that systemic administration of BCG promotes bone marrow emergency granulopoiesis, which is likely one of the early steps for trained immunity (15). Emergency granulopoiesis and trained immunity are postulated to be a continuum of the same effect cascade (16). In the BCG training model, a hepatic nuclear factor (HNF) family member HNF1 was identified as a crucial regulator of the transcriptional shift of myeloid cell development and function within the HSPC compartment in the bone marrow (15), while in a low-virulence strain of *C. albicans* (PCA2) training model, GM-CSF has been shown to play a crucial role in the functional reprograming of HSPCs and promoting HSPC mobilization to the spleen (17).

### 2.2 Tissue-specific imprinting

The tissue-specific feature of trained immunity is another recent focus. Recent studies found that local immunological and metabolic environments play an important role in establishing trained immunity. Tissue innate cells usually have longer lifespan, for example, mouse alveolar macrophages (AMs) have a half-life of 30 days, while the lung interstitial macrophage lives even longer (18). Yao et al. showed that respiratory viral infection induces long-lasting (4–16 weeks) memory of AMs, which is independent of monocytes or bone marrow progenitors. The priming of the memory AMs requires help from IFNγ secreted by effector CD8 T cells (19). In agreement with this, intranasally administered heat-killed Mycobacterium tuberculosis induces more robust production of proinflammatory cytokines compared to the systemically administrated ones, highlighting the superiority of tissue-specific training (20). While lipopolysaccharide (LPS) induces a long-term epigenetic immunotolerance memory in hematopoietic stem cells (21), and in monocyte/macrophages (22), Zahalka et al. found that intranasal exposure to LPS induces a pronounced AM memory response, where type 1 interferon signaling, fatty acid oxidation and glutaminolysis account for the enhanced reactivity upon pneumococcal challenge (23). The difference might be due to the fact that the intranasal exposure to LPS had the ability to train any lung-resident cell populations, including AM and other (non-AM) cell populations (e.g. resident structural, myeloid or lymphoid cell types), and thus to alter their responsiveness to S. pneumoniae challenge. This study underlined the importance of considering multiple cellular players and tissue-specific parameters when tissues are exposed to trained immunity agents. The circulating microbial metabolites, which reflect the alteration of intestinal microbiota after subcutaneous BCG vaccination, also leads to the training of AMs (24). Besides macrophages, epithelial cells, which constitute the first line of defense, have an essential role in the long-lasting response after exposure the external stimulus (4). Moreover, as most of the mucosal tissues have non-immune tissue-specific stem cells, which have an even longer lifespan (25–27), the direct imprinting of these cells result in the maintenance of long-term trained immunity. For example, the alteration of chromosomal accessibility in epithelial stem cells (EpSCs) of skin enables a prolonged memory upon acute inflammation. Aim2, which encodes an activator of the inflammasome, or its downstream effectors, caspase-1 and interleukin-1beta, is responsible for the memory, and the skin-resident macrophages or T cells are not required for this functional adaptation (28). Overall, the tissue-specific training underscores the mucosal administration of training agents.

### 2.3 Interferons (IFN) in imprinting

Notably, recent studies highlighted the important roles of interferons (IFNs), especially IFNγ, in the induction and maintenance of trained immunity (19, 29–33). It has been shown that BCG reprograms HSPC and confers protection against later Mycobacterium tuberculosis (Mtb) via the IFNγ pathway (30). However, if trained with Mtb, it reprograms HSPCs differently by switching IFNγ pathway to type I IFN/iron axis, which results in the induction of necroptosis in the myeloid lineage, and eventually impairs the trained macrophage immunity (29). In two other BCG trained models, IFNγ produced by CD4+ T cells or CX3CR1hi CD4+T cells imprints tissue myeloid and epithelial cells to generate prolonged and broad innate antiviral resistance (31, 32). Interestingly, for respiratory viral infection induced trained immunity, it is the IFNγ produced by CD8+ T cells that renders long-term memory of alveolar macrophages in the lung (19). Collectively, these findings demonstrate the profound impact of IFNγ on tissue-specific trained immunity, which could have direct implication in mucosal cancer therapies. Since both primary and metastatic cancers frequently target mucosal tissues, the innate and adaptive anti-cancer immunity at mucosal sites had the advantage of killing the tumor cells at an early stage. Indeed, this fits with the notion that IFNγ needs to be delivered at the right time to the right place to generate more effective anti-tumor immunity (34, 35).

All things considered, these studies and the studies shown below highlighted the essential role of cytokines in establishing and maintaining of trained immunity (**Table 1**). Induced by various of agents and secreted by T cells or myeloid cells, these cytokines, including type I and II IFNs, GM-CSF, HIF-1α/IL-1β, HNF1α&1β, and IL-12/15/18, might have direct effects on HSPC, myeloid cells and natural killer (NK) cells to furnish them prolonged memory.

**Table 1:** Cytokines that involved in the establishment of trained immunity.

Cytokines	Induced by	Secreted by	Imprinting cell types	refs
IFNγ	BCG; respiratory viral infection;	CD4+cells; CX3CR1hi CD4+T cells; CD8+ cells	HSPC; monocytes/macrophage	(19, 29–33)
Type I IFN	β-glucan	Not identified	granulopoietic progenitors/neutrophils	(36)
HIF-1α/IL-1β	Succinate; β-Glucan particles; heat-killed Mycobacterium tuberculosis	myeloid cells; monocytes; macrophage	myeloid cells; monocytes; macrophage	(10, 20, 37)
HNF1alpha;&1β	BCG	Not identified	HSPC; myeloid cells; granulocyte	(15)
GM-CSF	*C. albicans*	Not identified	HSPC	(17)
IL-12/15/18	IL-12/15/18-treated NK cells	Not identified	NK cells	(38–40)

IFN, interferon; HIF, hypoxia-inducible factor; IL, interleukin; HNF, hepatic nuclear factor; GM-CSF, Granulocyte-macrophage colony-stimulating factor; HSPC, hematopoietic stem and progenitor cell; NK, natural killer.

## 3 Trained immunity inducers in cancer immunotherapy

**Figure 1:**
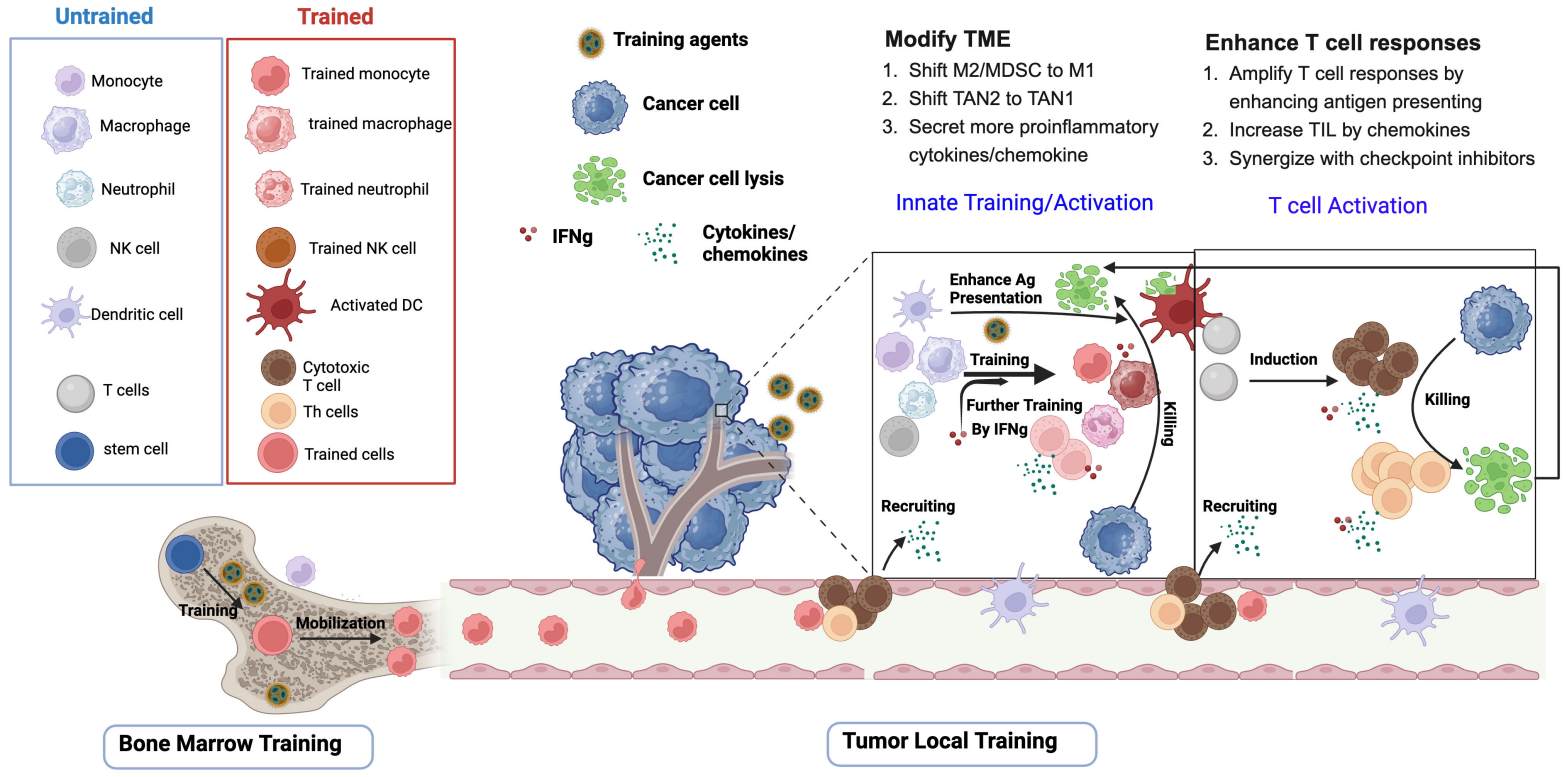
Trained immunity inducers modify TME and enhance T cell responses. Upon initial exposure to training agents (apply to all training agents), stem cells or hematopoietic progenitor cells in bone marrow, as well as a myriad of innate immune cells in the tumor microenvironment (TME), including monocytes, macrophages, neutrophils and natural killer cells, undergo epigenetic and metabolic programing and acquire trained features. Upon second stimulation, which could be the same training agents or the proliferation of the tumors, the trained innate cells and the proinflammatory cytokines such as interferons mediate tumor cell lysis through phagocytosis, ROS, and cytotoxicity. The progeny of stem cells in the bone marrow are also mobilized to the tumor. Trained innate inducers promote T cell induction through enhancing dendritic cell (DC)-mediated tumor antigen presentation. After capturing tumor-associated antigens in the tumor, DC elicit tumor-specific T cells either in the mucosal tissues or after migrating to the draining lymph nodes. The chemokines released by activated innate cells and T cells are responsible for recruiting more T cells to infiltrate the tumors. Importantly, IFNγ produced by T cells or activated innate cells further participates in the training of the innate cells in the TME.

Cancer immunotherapy holds great promise for inhibiting tumor growth and metastasis through manipulating the immune system. However, only suboptimal efficacy has been shown in many of the trials, as T cell responses are the main effector cells and the innate immune system is not efficiently mobilized (41). Agents, such as bacteria, fungi, and their components, as well as TLR agonists and cytokines, that are known to induce trained immunity, are therefore named trained immunity inducers, and have been shown to reduce tumor progression and control tumor metastasis. The utilization of these trained immunity inducers alone, or in combination with other cancer therapy strategies, can help to induce robust and long‐lasting antigen‐non-specific immunity, which shifts the immunosuppressive TME to a proinflammatory antitumor state, and facilitates the establishment and maintenance of antigen-specific adaptive immunity (**Figure 1**). A range of trained immunity inducers, for example BCG and β-glucan, have been used to fight against cancer in preclinical and clinical studies (**Tables 2**–**4**).

**Table 2:** Trained immunity in cancer immunotherapies (Pre-clinical studies).

TI inducer/route	Tumor	Innate cells involved	Mechanisms	Refs
BCG/intravesical	MB49 orthotopic bladder cancer	Not identified	Activation, reduced exhaustion, and ultimately enhanced effector function of tumor-specific T cells, esp.CD4+T producing IFNg	(42)
BCG-STING/intravesical	Orthotopic urothelial cancer	M1-like myeloid cells	IFN-beta, epigenetic program of proinflammatory cytokine, glycolysis	(43, 44)
BCG/sc	HCCs	M1 macrophages	T cells, trained immunity, IFN-gamma	(33)
B-Glucan/IP	B16-F10 & other cancers	Granulopoietic progenitors/neutrophils	Type I IFN signaling and ROS are causally linked	(36)
β-Glucan particles/IP	Orthotopic pancreatic cancer	CCR2+ myeloid cells	Enhanced phagocytosis and ROS-mediated cytotoxicity	(45)
β-Glucan particles/IP&IO	Orthotopic pancreatic cancer	CD14+myeloid cells	Increase total immune cell infiltration in the PC TME; enhanced phagocytosis and cytotoxicity	(46)
β-Glucan particles/IP	Lung metastases models	lung interstitial macrophage	Macrophage phagocytosis and cytotoxicity through sphingosine-1-phosphate (S1P)-mediated mitochondrial fission pathway	(47)
β-Glucan –Ecoli+OVA/SC	B16-OVA	Monocytes/Macrophages and DCs	Higher TNFalpha;, CD80 expression in trained cells; enhanced adaptive immunity	(48)

**Table 3:** Trained immunity in cancer immunotherapies (Current clinical studies).

TI inducer/route	Tumor	Mechanisms	Refs
BCG/intravesical	NMIBC	Enhanced cytokine expression upon LPS stimulation *in vitro* in monocytes; autophagy is essential for BCG and β-glucan training of monocytes	(49)
BCG/intravesical+ Cancer vaccine	NMIBC	Vaccine-specific T cell in urine in BCG treated group	(50)
BCG/intravesical	NMIBC	Higher post-BCG:pre-BCG ratio of IL-12 in monocyte associated with longer time to recurrences.	(51)
BCG/intravesical	NMIBC	Enhanced production of TNF and IL-1β in circulating monocytes upon heterologous ex vivo stimulation; enhanced inflammasome activity; reduced respiratory infections	(52)
BCG/intravesical +checkpoint inhibitor	Neuroendocrine tumor	elevated monocyte-derived interleukin-6 and interleukin-1β production capacity; the feasibility to combine both treatment	(53)

NMIBC, non-muscle–invasive bladder cancer.

**Table 4:** On-going clinical trials involved trained immunity in cancer immunotherapies.

TI inducer/route	Tumor	Treatment	Sponsor/Location	ClinicalTrials.gov ID
BCG/percutaneous	HPV	9vHPV vaccine plus BCG	Medicine Invention Design, Inc./Rockville, Maryland, USA	NCT02403505
Various of TI agents	Thyroid/Colon Carcinoma	Myeloid cells treated Ex vivo	Radboud University Medical Center/Nijmegen, Gelderland, Netherlands	NCT05280379
LPS	Solid Cancer	TLR4+ Blood Cell Characterization	Hospices Civils de Lyon, France	NCT06131775

HPV, human papillomavirus; LPS, lipopolysaccharide; TLR4, Toll-like receptor 4.

### 3.1 BCG

Bacille Calmette-Guérin (BCG), a vaccine derived from live attenuated Mycobacterium bovis, is the most administered vaccine in human history (54). It has been used as the primary tuberculosis vaccine since the 1920s. It induces both specific immune responses against tuberculosis and non-specific beneficial effects against other unrelated infections and cancer.

#### 3.1.1 BCG in non-muscle–invasive bladder cancer (NMIBC)

BCG stimulates anti-tumor immune response, which has been harnessed for urothelial cell carcinomas, especially NMIBC treatment. BCG treatment was first used as adjuvant immunotherapy against neoplasia in 1976 (55). Despite various therapy strategies developed, intravesical BCG remains the standard-of-care treatment for high risk NMIBC (56). However, the mechanisms of BCG-induced immunity in NMIBC are not yet fully understood. Recently, a growing body of evidence indicates that protection against NMIBC offered by BCG was at least partially attributed to trained immunity (49, 52, 57). Delineating the mechanism of this antigen-agnostic effect of BCG is important for the clinical application. Trained immunity is elicited by epigenetic and metabolic reprogramming of the innate cells. Buffen et al. identified autophagy is a key player in trained immunity induced by BCG (49). Blocking autophagy inhibits H3K4 trimethylation of monocytes, a key processing of epigenetic reprogramming (49). Moreover, single nucleotide polymorphisms (SNPs) in the autophagy genes ATG2B (rs3759601) and ATG5 (rs2245214) affect the training effects of BCG. In clinical studies, Van Puffelen et al. observed that circulating monocytes had increased production of TNF and IL-1β upon heterologous ex vivo stimulation after intravesical BCG treatment of patients with NMIBC. Further *in vitro* studies identified enhanced inflammasome activity in BCG-treated individuals (52). In another cohort of NMIBC patients treated with BCG, patients with innate immune memory (based on IL-12 ratios) had significantly longer time to tumor recurrence (disease-free survival) compared to those without innate immune memory (51).

To improve the BCG-mediated trained immunity levels, a recombinant BCG was constructed to release high levels of c-di-AMP, an agonist recognized by stimulator of interferon genes (STING). Compared to wild type BCG, the BCG-STING demonstrates higher trained immune responses, including eliciting IFNβ release in macrophages, inducing epigenetic activation marks in proinflammatory cytokine promoters, shifting monocyte metabolomic profiles toward glycolysis, and reprograming myeloid cells toward M1-like (43, 44). Importantly, improved antitumor efficacy can be achieved by the BCG-STING in preclinical bladder cancer model, suggesting that the enhanced trained immunity contributes to the better inhibition of tumor growth (43, 44).

BCG therapy can not only avert a suppressive TME, but also improve T cell responses. In murine MB49 orthotopic model of bladder cancer, weekly intravesical treatments of BCG leads to long-term tumor-specific immunity. The tumor eradication is predominantly CD4 T cell dependent, and the major effect of BCG is to enhance the effector functions of tumor-specific CD4 T cells, specifically IFN-γ production (42). Derre et al. showed that intravesical BCG therapy affects the local immunostimulation status and facilitates the tumor-specific T-cell infiltration into the bladder of the patients with NMIBC (50). As tumor-specific T cell immunity is critical for the tumor elimination, the increased vaccine-specific T cells in the bladder upon BCG stimulation indicates that cancer vaccines can be improved by combining with trained immunity.

#### 3.1.2 BCG in hepatocellular carcinoma (HCC)

Besides NMIBC, BCG has demonstrated therapeutic effects on liver metastases and trained immunity at least partially participates in tumor rejection. In orthotopic HCC mouse models, one subcutaneous injection of BCG in HCC-bearing mice leads to more effective anti-tumor effects than the anti-PD-1 therapy does. BCG treatment results in the recruitment of M1 macrophages and T cells to the TME and enhances IFN-γ production. It seems that similar mechanisms in BCG treated bladder cancer apply to BCG-treated HCC as well. Evidence of the involvement of trained immunity and T cell responses in reducing tumor burden are shown by blockage studies. BCG’s anti-HCC effects can be abolished by depletion of T cells, trained immunity, and IFNγ function (33). Specifically, the authors showed that blocking trained immunity by metformin, which can inhibit the trained immunity property of BCG by reducing the production of IL-6 and Tumor necrosis factor-*α* (TNF-*α*), abrogates the anti-HCC effect of BCG (33). In a patient with gastric neuroendocrine carcinoma and liver metastases, the BCG vaccine combined with PD-1 inhibitor nivolumab leads to mild progression of the primary tumor and no progression of the metastases, suggesting the feasibility of using trained immunity inducers as an adjuvant to immune checkpoint therapy (53). BCG has also been used to treat other malignancies, such as lung, melanoma, leukemia, and lymphoma. However, whether trained immunity contributed to the treatment outcome is not clear (58).

#### 3.1.3 BCG prevents infections in immune compromised malignancy patients

Patients with malignancies, especially hematological/lymphoid malignancies, are often immune compromised, which leads to higher risk of recurrent infections. As infectious complications are a major cause of morbidity and mortality in hematological malignancies, trained immunity might be a promising strategy in the management of recurrent infections in these patients. BCG has been shown to induce non-specific cross-protection against various of pathogens such as Candida albicans, Staphylococcus aureus, as well as some DNA and RNA viruses (59). MV130, a suspension of heat-inactivated whole cell bacteria, has been shown to induce trained immunity, which protects against experimental viral respiratory infections in mouse models (60). Thus, BCG or other trained immunity inducers, for example MV130, could be used to ameliorate the increased risk of infectious complications in the immune compromised patients (61). Indeed, MV130 was found to be able to reduce the rate of recurrent respiratory tract infections and enhance humoral immune responses in patients with hematological malignancies (62).

In summary, BCG-induced trained immunity contributes to the control of various of cancers including NMIBC, liver and other metastases. Currently, genetically modified BCG candidates expressing molecules that confer stronger protection against NMIBC are under investigation (63).

### 3.2 β-glucan

The beneficial properties of orally administrated β-glucans have long been recognized. The proposed mechanisms of action in cancer therapy include enhanced antitumor T cell immunity, as well as the modification of the innate arm of the immune responses, for example stimulation of suppressive myeloid subsets, induction of inflammatory monocytes, and enhancement of complement receptor 3-dependent cellular cytotoxicity in neutrophils (64, 65). Yeast-derived β-glucan, a natural compound with immune-stimulatory and immunomodulatory potential, has recently been shown to induce trained immunity and have the ability to reverse the epigenetic states of LPS-induced immunological tolerance (66, 67). Two forms of β-Glucans, soluble fiber β-Glucan and whole β-Glucan particles (WGP), are used to investigate the role of trained immunity involved in cancer immunotherapy:

#### 3.2.1 β-glucan in pancreatic ductal adenocarcinoma (PDAC) and lung tumor metastasis

PDAC is characterized by infiltration of immunosuppressive cell subsets including tumor-associated macrophages (TAMs), regulatory T-cells (T-regs), and myeloid-derived suppressor cells (MDSCs), and most importantly, not enough infiltration of activated anti-tumor immune cells (68). β-glucan has been used to target the immune cells within the TME of PDAC. Daily oral β-glucan therapy elicits heightened TNF-α production from CD86+ monocytes in newly diagnosed PDAC patients who had undergone surgical ablation (46). In the orthotopic pancreatic cancer (PC) murine model, β-glucan enhances immune cell infiltration to the PC and augments the trained properties of tumor-infiltrating myeloid cells (46). The combination of surgical ablation with oral β-glucan administration reduces local and distant tumor burden and prolongs survival independent of adaptive immune responses (46).

The hollow structure of particulate β-glucan (WGP) makes it an ideal delivery vesicle. WGP is used to polarize myeloid cells toward antitumor phenotypes. Geller et al. observed that IP injected whole β-Glucan particles or the macrophages with WGP have pancreas-tropism and induce an influx of CCR2+ myeloid cells that show a phenotype of trained immunity, such as enhanced phagocytosis and ROS-mediated cytotoxicity to tumor cells. Furthermore, trained immunity contributes to tumor regression and prolonged survival in murine orthotopic models of pancreatic cancer (45). These studies suggested that β-glucan can avert PC’s notoriously immunosuppressive TME and turn cold PC tumors into hot ones. Indeed, β-glucan has been shown to synergize with anti-PD-L1 mAb therapy to prolong survival in murine PDAC models (45).

Tumor metastasis is the leading cause of cancer-related deaths, and myeloid cells in the pre-metastatic niche provide a supportive environment for tumor survival and growth (69). WGP effectively controls tumor metastasis by eliciting a trained immune response in lung interstitial macrophages (IMs), which can be activated upon exposure to tumor cells and tumor-derived soluble factors (47). Both HIF-1α and IL-1β–IL-1R signaling have been implicated in β-glucan-induced trained immunity. However, Ding et al. demonstrated that this signaling is not essential for WGP-mediated lung interstitial macrophage trained immunity and *in vivo* cancer metastasis control (47). Instead, WGP augments lung interstitial macrophage phagocytosis and cytotoxicity through the sphingosine-1-phosphate (S1P)-mediated mitochondrial fission pathway, which is responsible for the inhibition of tumor metastasis and prolonged tumor-free survival (47).

#### 3.2.2 β-glucan in OVA, HPV-E7 model antigen tumor models

Tumor antigens have been embedded with β-glucan to probe the interaction between trained immunity and antigen-specific T cell induction. In a recent study, Chen et al. developed a cancer vaccine with the attachment of model antigen OVA and β-glucan onto the surface of an inactivated probiotic Escherichia coli (48). Both the bacteria and β-glucan can act as trained immunity inducers in mice. The combined vaccine efficiently induces trained immunity of macrophages, as evidenced by the enhanced numbers of proinflammatory macrophages at the injection sites, higher expression of TNF‐α after secondary stimulation and increased phagocytosis of tumor cells *in vitro* (48). The vaccine-induced trained immunity, for example, the significantly increased expression of proinflammatory cytokines, might direct the adaptive immunity toward a more effective response against tumors. Indeed, the author showed that in 4T1 tumor-bearing mice undergoing surgical tumor resection, the mice that received the combined vaccine significantly enhanced the ratios of CD8+ central memory and effector memory T cells in blood compared with those of naïve mice, suggesting the efficient induction of immunological memory, which might prevent postoperative tumor recurrence (48). In an HPV-E7 model antigen study, a nano-cancer vaccine embedded with trained immunity trainers, muramyl Dipeptide (MDP) and β-glucan, showed enhanced protective efficacy against TC-1 tumor (70). Accompanied by increased production of trained immunity signature cytokines IL-1β, IL-6, and TNF-α *in vitro* and *in vivo* upon secondary homologous or heterologous stimulation, E7-specific IFN-γ-expressing CD8+T cells are significantly higher in the vaccinated group with trained immunity trainers than the groups that received vaccine without trainers or trainers alone (70). These studies suggest that β-glucan might play a role in promoting the interaction between trained immunity and T cell responses for tumor control. However, due to the lack of proper controls in these studies, more evidence is needed to confirm the conclusion.

#### 3.2.3 Neutrophils and type I IFN in β-glucan-induced trained immunity

Though myeloid cells are the major components of tumor immune cell infiltrate (71), neutrophils can infiltrate tumor as well. The tumor-associated neutrophils can be classified into two functional opposite types: TAN1, induced by type I interferons, is cytotoxic and anti-tumorigenic, while TAN2, elicited by TGF-β, promotes tumor progression (72). Kalafati et al. found that β-glucan, as a trained immunity inducer, modifies the TME through shifting the neutrophils from TAN2 to TAN1. The transcriptome of neutrophils, but not monocytes, from β-glucan-trained mice shows significant differences compared to non-trained controls. β-glucan pretreated mice suppress tumor growth independent of adaptive immunity in a B16F10 melanoma model. The long-term anti-tumor TAN1 effect is attributed to transcriptomic and epigenetic rewiring of granulopoiesis after β-glucan training (36). In contrast to BCG-training, where monocytic lineage (monocytes/macrophages) and IFNγ are essential for the induction of trained immunity, β-glucan elicited trained immunity seems relying on granulopoietic progenitors/neutrophils and type I IFN (36). Though neutrophils are also implicated to exert anti-bladder cancer effect in a murine BCG model (73).

### 3.3 Nanobiologics and epigenetic regulators

Nanomaterials, such as apolipoprotein A-1(apoA1), the main protein constituent of high-density lipoprotein (HDL), have affinity for myeloid cells and their progenitors through their ATP-binding cassette transporter A1/G1 (74). Peptidoglycans and their derivatives can induce trained immunity via the activation of Nucleotide-binding oligomerization domain-containing protein 2 (NOD2) receptor (12). Priem et al. bioengineered the peptidoglycan derivatives onto the surface of HDL to retain the trained immunity-inducing structure and high bone marrow avidity (75, 76). The engineered biologics provoke trained immunity-mediated anti-tumor activity after being intravenously administrated in a mouse B16F10 melanoma model (76). The trained immunity mechanisms include enhanced proliferation and metabolism of bone marrow progenitors and higher levels of myeloid cells with increased cytokine response upon heterologous stimulation. When combined with checkpoint inhibitor treatment, the nanobiologics primes the immune system’s responsiveness to checkpoint blockade therapy (76).

As epigenetic programing is one of the mechanisms of inducing trained immunity, direct targeting of epigenetic modulators, such as histone deacetylases (HDACs), might induce trained immunity to inhibit tumor growth. Indeed, in a mouse breast cancer model, HDAC inhibitor TMP195 treatment averts the suppressive tumor microenvironment, reduces tumor burden and pulmonary metastases (77).

### 3.4 Cytokine-induced memory-like natural killer (NK) cells

NK cells can acquire features of trained immunity after a wide range of stimuli, such as cytomegalovirus (CMV) infection or pro-inflammatory cytokine stimulation (38, 78). NK cells are especially important for killing tumor cells that have suppressed expression of class I MHC molecules, preventing them from being killed by adaptive CD8 T cells (79). In humans, the presence of class I HLA-E is detected by NK receptors CD94/NKG2/A, B, C and this turns them off (80). Of note, cytokines IL-12/15/18 stimulate murine and human NK cells to secret more IFNγ (38, 39, 81, 82) and hold great promise for cancer immunotherapy. Indeed, IL-15, discovered by Tom Waldmann (83, 84) is absolutely essential for NK cell development, as these are completely absent in IL-15 KO mice (85). Numerous preclinical and clinical studies were carried out and adoptive transfer of IL-12/15/18-trained NK cells show superior antitumor properties against established murine tumors including leukemia, multiple myeloma, ovarian cancer, HCC, melanoma with or without other therapy strategies (reviewed in (40)). Moreover, a superactive form of IL-15 (N803) was just recently licensed by the FDA to treat certain cancers. Interestingly, similar training mechanisms of myeloid cells or neutrophils might also apply to IL-12/15/18-trained NK cells as well (40). Currently, the combination of cytokine-trained NK cells with other cancer immunotherapy strategies such as CAR-NK cells (86) or checkpoint inhibitors are under investigation.

## 4 Repurposing of trained immunity inducers from other fields

These studies pave the way for exploiting trained immunity for cancer treatment, both as monotherapy and in combination with other immunotherapeutic strategies. To harness the power of trained immunity more efficiently, there is a pressing need to expand the repertoire for more potent inducers. TLR agonists, cytokines, and viruses can also induce trained immunity as shown in other disease models, since trained immunity can be initiated by the direct recognition of Microbe-Associated Molecular Patterns (MAMPs) by Pattern Recognition Receptors (PRRs) or by cytokines released during vaccinations or infections. The list of these inducers has been summarized in the reviews (87, 88). Repurposing of these training agents would pave a new path for cancer immunotherapy. Some of these agents, for example, TLR9 agonists CpG (89) and IL-15 (90) have been utilized for cancer immunotherapy, and demonstrated insufficient efficacy as a single agent. It is not clear whether and how trained immunity is involved in tumor suppression for these agents, although IL-15 at least clearly expands NK cells enormously.

Given that the TME in cancer patients is profoundly affected, head-to-head comparisons of these training agents in preclinical models are necessary. In line with this, delineating the induction mechanism of each training agent, and combining the ones with different mechanisms might lead to synergy and thus better cancer control.

## 5 Considerations and challenges of exploiting trained immunity for cancer immunotherapy

When exploiting trained immunity in cancer immunotherapies, several crucial considerations come into play. First and foremost, we should be cautious that there are significant overlaps between mechanisms and pathways for cancer cell survival and those for the induction of trained immunity. For example, mTOR- and HIF-1alpha;-mediated aerobic glycolysis is one of the central hallmarks of trained immunity metabolism (10), but this is also a major metabolic pathway for cancer cells. A similar situation exists for mevalonate metabolism. The dual nature of these pathways provides challenges and cautions for manipulating these pathways in training innate cells for cancer immunotherapy (91). Moreover, one consideration is that the main characteristic of trained immunity is a pro-inflammatory environment, which is unfavorable in a cancer process. Second, more rigorous preclinical studies to test the mechanisms and anti-tumor efficacies of different trained immunity inducers are required. BCG and β-glucan are the most frequently used trained immunity inducers for cancer immunotherapy. However, trained immunity has various of flavors. The mechanisms and pathways involved, as well as the anti-cancer efficacies are different. To achieve the best anti-tumor results, tailoring the optimal inducers for each type of tumor might be needed. In the case of combining trained immunity with immunotherapy, the order also matters and needs to be optimized. Training the system first might alter the suppressive TME and induce PD-1, which improves the immunotherapy efficacy. On the other hand, training the system after immunotherapy might prevent the development of metastasis. Third, the choice of the appropriate routes and doses is of great importance. The route of administration affects the magnitude of trained immunity: for examples, intravenous (IV) administration of BCG is more effective than intradermal or subcutaneous routes. One plausible reason is that IV administration allows the BCG to more easily gain access to the BM compared to the other routes (57). Importantly, local training in tissues highlights the importance of mucosal route delivery of the training agents. The best way to achieve trained immunity might need the engagement of both BM and local tissues. The newly proposed concept of “integrated organ immunity” by Pulendran describes the interaction among the innate and adaptive immune systems and non-hematopoietic cells in tissues, which induces long-lasting protection against pathogens independent of specific antigens (92). Strategies to apply integrated organ immunity for tumor immune therapy warrant further investigation. Another important question is the dose. The doses reported to activate the trained immunity with antitumor properties varied a lot. For BCG, from 1–5X10^6 CFU/dose to 1–10 μg/ml (15, 20, 33, 43, 49), and for β-glucan: from 10 ng/ml (10, 49), 20 μg/mL (47), to100 μg/mL (20) has been used. Last but not least, it is crucial to establish the safety and efficacy in human subjects. More clinical trials need to be conducted to assess the overall impact on antitumor immunity in cancer patients by using the optimized regimens to induce trained immunity discovered in preclinical models.

## 6 Future directions

Despite significant advancements and implementation of trained immunity in cancer immunotherapy, many obstacles remain to be a barrier for successful translation to patient treatment. The following outstanding questions need to be addressed urgently. 1). Which trained immunity inducers or combinations lead to the optimal and long-term cancer remission? 2). What trained immunity mechanisms or biomarkers are associated with cancer control? 3). Which delivery strategies are ideal for the induction of localized- and/or systemic- trained immunity to prevent cancer growth and metastases? More efforts are needed to fill the knowledge gap and to deploy trained immunity for the development of broad-spectrum anti-cancer immunotherapy.

Search strategy and selection criteria:

Data for this Review were identified by searches of PubMed from relevant articles using the search terms “trained immunity”, and “cancer immunotherapy”.

## Author contributions

YS: Conceptualization, Writing – original draft, Writing – review & editing. JB: Writing – review & editing.
